# Non-typeable pneumococci circulating in Portugal are of *cps* type NCC2 and have genomic features typical of encapsulated isolates

**DOI:** 10.1186/1471-2164-15-863

**Published:** 2014-10-06

**Authors:** Débora A Tavares, Alexandra S Simões, Hester J Bootsma, Peter WM Hermans, Hermínia de Lencastre, Raquel Sá-Leão

**Affiliations:** Laboratory of Molecular Microbiology of Human Pathogens, Instituto de Tecnologia Química e Biológica (ITQB) António Xavier, Universidade Nova de Lisboa (UNL), Oeiras, Portugal; Laboratory of Paediatric Infectious Diseases, Radboud University Nijmegen Medical Centre, Nijmegen, the Netherlands; Crucell – Johnson and Johnson, Leiden, the Netherlands; Laboratory of Molecular Genetics, ITQB, UNL, Oeiras, Portugal; Laboratory of Microbiology and Infectious Diseases, The Rockefeller University, New York, NY USA

## Abstract

**Background:**

Pneumococcus is a major human pathogen and the polysaccharide capsule is considered its main virulence factor. Nevertheless, strains lacking a capsule, named non-typeable pneumococcus (NT), are maintained in nature and frequently colonise the human nasopharynx. Interest in these strains, not targeted by any of the currently available pneumococcal vaccines, has been rising as they seem to play an important role in the evolution of the species. Currently, there is a paucity of data regarding this group of pneumococci. Also, questions have been raised on whether they are true pneumococci. We aimed to obtain insights in the genetic content of NT and the mechanisms leading to non-typeability and to genetic diversity.

**Results:**

A collection of 52 NT isolates representative of the lineages circulating in Portugal between 1997 and 2007, as determined by pulsed-field gel electrophoresis and multilocus sequence typing, was analysed. The capsular region was sequenced and comparative genomic hybridisation (CGH) using a microarray covering the genome of 10 pneumococcal strains was carried out. The presence of mobile elements was investigated as source of intraclonal variation. NT circulating in Portugal were found to have similar capsular regions, of *cps* type NCC2, i.e., having *aliB*-like ORF1 and *aliB*-like ORF2 genes. The core genome of NT was essentially similar to that of encapsulated strains. Also, competence genes and most virulence genes were present. The few virulence genes absent in all NT were the capsular genes, type-I and type-II pili, choline-binding protein A (*cbpA*/*pspC*), and pneumococcal surface protein A (*pspA*). Intraclonal variation could not be entirely explained by the presence of prophages and other mobile elements.

**Conclusions:**

NT circulating in Portugal are a homogeneous group belonging to *cps* type NCC2. Our observations support the theory that they are *bona-fide* pneumococcal isolates that do not express the capsule but are otherwise essentially similar to encapsulated pneumococci. Thus we propose that NT should be routinely identified and reported in surveillance studies.

**Electronic supplementary material:**

The online version of this article (doi:10.1186/1471-2164-15-863) contains supplementary material, which is available to authorized users.

## Background

Pneumococcus is a major human pathogen, causing a wide range of infections from otitis media to bacteraemia and meningitis. Its main virulence determinant is a polysaccharide capsule that surrounds pneumococcal cells, providing protection against phagocytosis [1]. Together with colony morphology, susceptibility to optochin, and bile solubility, assignment of a serotype (based on the capsular type) has been traditionally the ultimate assay to identify pneumococcus [2]. To date, more than 95 serotypes have been described and, with the exception of type 37, the genes responsible for the expression of the capsule are located in the chromosome between the *dexB* and *aliA* genes (capsular region) [1, 3]. The pneumococcal capsule is also the target of all currently available pneumococcal vaccines [4].

Pneumococci lacking a polysaccharide capsule are known to exist in nature and are frequent inhabitants of the upper respiratory tract of humans [5]. Although these isolates, often named non-typeable pneumococcus (NT), are mostly asymptomatically carried in the nasopharynx, they have also been associated with conjunctivitis outbreaks and sporadically associated with other disease manifestations including invasive disease [6–9]. Studies have suggested, using a combination of phenotypic and genotypic methods, that some of these isolates are *bona-fide* pneumococci and share common properties with encapsulated pneumococci [5, 10]. Also, in vitro studies with non-encapsulated pneumococci have shown that these strains display increased adherence to epithelial tissue, increased capacity for biofilm formation, and are highly transformable [11–13]. Hence, high carriage rates combined with high transformability rates may provide NT with the features needed to play an important role in the evolution of pneumococcus as recently proposed by Chewapreecha, *et al.* [14].

In a previous study, we have described the population structure of NT strains in Portugal and identified major lineages associated with them [5]. In parallel, others have identified the same lineages in circulation in other geographical settings and the capsular region of NT has been characterised [10, 15–17]. Based on the capsular region, NT have been proposed to be divided in two groups: Group I includes isolates with a disrupted or non-functional capsular locus and Group II includes isolates with genes not found in conventional capsular types [17]. Group II NT have been proposed to be further divided into *cps* types NCC1, when isolates have the *pspK* gene (pneumococcal surface protein Korea, also referred to as *nspA*, non-typeable pneumococcal surface protein A), encoding for a novel pneumococcal surface protein with several features suggesting a role in cell adhesion and enhanced colonisation, and NCC2, when isolates have both the *aliB*-like ORF1 and *aliB*-like ORF2 genes, predicted to encode for lipoproteins [15–18]. A *cps* type NCC3 has also been described for isolates with *aliB*-like ORF2 but not *aliB*-like ORF1, but these were shown not to be pneumococci [15].

The observation that several distinct clonal lineages lacking the capsule operon have been in circulation for decades and are not derived from encapsulated strains has raised the question of how different is the genome of these strains compared to encapsulated pneumococci [5, 8]. The aim of this study was to characterise a carriage collection of NT circulating in Portugal in a period of 11 years to obtain insights into the genetic basis of non-typeability and their genomic content and diversity.

## Results

### Capsular region of NT

**Figure 1:**
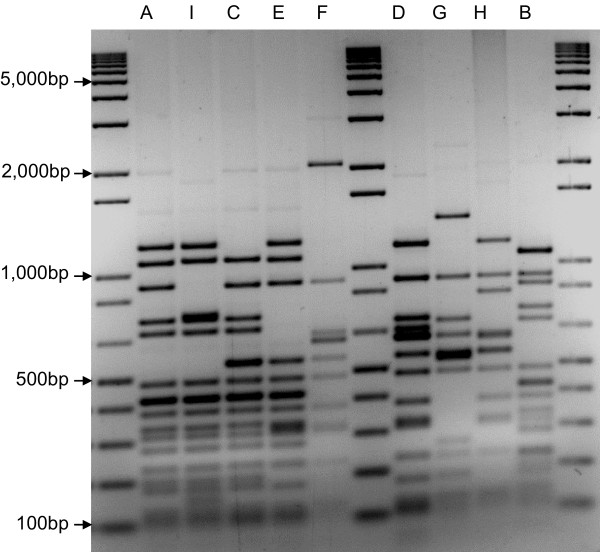
**RFLP patterns of the capsular region of NT strains with HinfI.** Capital letters in lanes refer to an arbitrary pattern designation.

**Figure 2:**
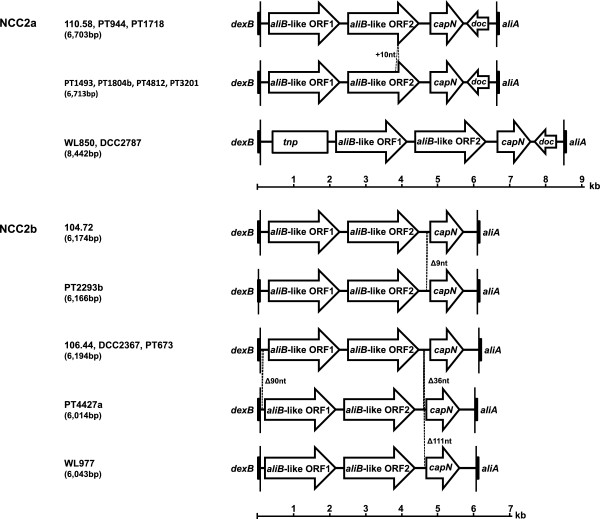
**Schematic representation of the capsular region of NT strains.** NCC2a and NCC2b refer to a classification of *cps* types proposed by Park, *et al.*[15]. Published sequences of strains 110.58 [GenBank:AY653211.1], 104.72 [GenBank:AY653210.1], and 106.44 [GenBank:AY653209.1] are shown for comparative purposes [17]. *capN* and *doc* indicate *capN*-like and *doc*-like regions, respectively.

To obtain insights into the genetic basis of non-typeability, the capsular region was characterised for a set of 42 NT strains representative of the lineages detected in cross-sectional colonisation studies conducted in Portugal among children between 1997 and 2007 (Table 1). Amplification of this region yielded, in all strains, a fragment of 6,000-8,500 bp. To investigate the heterogeneity of the capsular region, restriction fragment length polymorphism (RFLP) patterns were determined by digestion with HinfI. Nine different patterns could be distinguished after digestion with HinfI (Figure 1, Table 1). We then selected 13 isolates, representative of the different capsular RFLP patterns found in each CC, for sequencing. The findings are summarised in Figure 2 that shows a schematic organisation of the locus compared to strains previously described by Hathaway, *et al*. [17]. All strains had *aliB*-like ORF1, *aliB*-like ORF2, and *capN*-like regions; eight had the *doc*-like region between *capN*-like and *aliA*. Based on the classification previously proposed by Park, *et al*. [15], the strains were therefore classified as belonging to *cps* type NCC2a (eight isolates containing the *doc*-like region) or NCC2b (the remaining five isolates). Of the eight strains belonging to *cps* type NCC2a, two had an insertion of a *tnp* region of ~1.7 kb between *dexB* and *aliB*-like ORF1 previously described [15, 16].

**Table 1:** **Study collection and characteristics of the strains**

CC ^a^	Strain	Year	PFGE	MLST	Antibiotype (non susceptible to) ^b^	CSP/ComD ^c^	Capsular region RFLP	Capsular region sequenced	Analysis by CGH
344	PT944	2001	NT1	344	PG, Ery, Da, Tet, SXT	2/2	A	Yes	Yes
	LGST142	2000	NT1	344	Ery, Da, Tet, SXT	2/2	A	No	No
	PT191	2001	NT1	344	PG, Ery, Da, Tet, SXT	2/2	A	No	No
	PT3412b	2002	NT1	344	Ery, Da, Tet, SXT	2/nd	A	No	No
	PT998	2001	NT1	344	PG, Ery, Tet, SXT	2/nd	A	No	No
	LGST214	2000	ND	344	Ery, Tet, SXT	2/nd	A	No	No
	DCC2367	1999	NT1	344	PG, Tet, SXT	2/2	F	Yes	No
	PT389	2001	NT1	344	PG, Tet, SXT	2/nd	F	No	No
	PT4427a	2002	NT1	344	PG, Ery, Tet, SXT	2/nd	H	Yes	No
	WL212	2001	NT1	1619	PG, Ery, Da, SXT	2/2	A	No	Yes
	PT5899	2007	NT1	5220	PG, Ery, Tet, SXT	2/nd	nd	No	Yes
	DCC635	1997	NT2	344	PG, Ery, Da, Tet, SXT	2/2	A	No	Yes
	WL992	2002	NT3	344	PG, Ery, Da, Tet, SXT	2/2	A	No	Yes
	PT2987	2002	NT4	344	PG, Ery, Da, Tet, SXT	2/nd	A	No	No
	PT2293b	2001	NT4	344	PG, Ery, Da, Tet, SXT	2/2	E	Yes	Yes
	PT6317	2007	NT5	344	PG, Ery, Da, Tet, SXT	2/nd	nd	No	Yes
	PT5838b	2007	NT6	344	Ery, Da, Tet, SXT	2/nd	nd	No	Yes
	WL1514	2003	NT7	344	PG, Ery, Da, Tet, SXT	2/2	A	No	Yes
	PT6318	2007	NT7	4586	PG, Ery, Da, Tet, SXT	2/nd	nd	No	Yes
	PT5269	2006	NT8	344	PG, Ery, Da, Tet, SXT	2/nd	nd	No	Yes
	DCC2879	1999	NT9	897	PG, Ery, Da, Tet, SXT	2/2	A	No	Yes
	PT1571b	2001	NT10	344	PG, Ery, Da, Tet, SXT	2/2	A	No	Yes
	PT5727	2006	NT11	344	PG, Ery, Da, Tet, SXT	2/nd	nd	No	Yes
	PT5082a	2003	NT22	344	PG, Ery, Da, Tet, SXT	2/nd	I	No	No
	WL598	2001	NT25	344	PG, Tet, SXT	2/nd	F	No	No
	DCC1795	1998	NT26	1541	PG, Ery, Da, Tet, SXT	2/2	A	No	Yes
	DCC2435p	1999	ND	344	Ery, Da, Tet, SXT	2/nd	A	No	No
1156	PT268	2001	NT21	1156	PG, Ery, Da, Tet, SXT	1/nd	A	No	No
	PT6210	2007	NT21	4583	PG, Ery, Da, Tet, SXT	1/nd	nd	No	Yes
	PT2687b	2001	NT22	1156	PG, Ery, Da, Tet, SXT	1/nd	A	No	No
	PT5561	2006	NT22	1156	PG, Ery, Da, Tet, SXT	1/nd	nd	No	Yes
	PT4014	2002	NT22	1153	PG, Ery, Da, Tet, SXT	1/1	C	No	Yes
	PT4222	2002	NT24	1156	PG, Ery, Da, Tet, SXT	1/1	A	No	No
	PT5002	2003	NT24	1156	Ery, Da, Tet	1/1	A	No	Yes
	PT1493	2001	NT24	1617	PG, SXT	1/1	A	Yes	Yes
	WL352.1	2001	NT24	1703	PG, SXT	1/1	A	No	Yes
	PT3201	2002	NT24	1153	PG, Ery, Da, Tet, SXT	1/1	C	Yes	Yes
	PT6209b	2007	NT24	4583	PG, Ery, Da, Tet	1/nd	nd	No	Yes
	PT2322	2001	ND	1153	PG, Ery, Da, Tet, SXT	1/nd	C	No	No
320	PT1804b	2001	NT19	888	PG, SXT	1/1	A	Yes	Yes
1540*	PT1718	2001	NT12	1540	SXT	1/4	A	Yes	Yes
1278*	PT4812	2003	NT22	1278	PG, SXT	1/1	A	Yes	Yes
941	DCC2787	1999	NT13	941	SXT	2/2	B	Yes	Yes
	WL165b	2001	NT13	1704		2/2	B	No	Yes
	DCC2648	1999	NT14	941	SXT	2/2	B	No	Yes
448	WL850a	2002	NT15	448		2/2	B	Yes	Yes
	WL1084	2002	NT15	448		2/2	B	No	No
	PT2417	2001	NT15	448	PG, SXT	2/nd	B	No	No
	WL108	2001	NT16	448		2/nd	nd	No	Yes
1618	PT673	2001	NT17	1618	PG, Ery	1/1	D	Yes	Yes
	WL402.1b	2001	NT17	1618	PG, Ery, Da, Tet, SXT	1/1	D	No	Yes
1705*	WL977	2002	NT23	1705	PG, SXT	1/1	G	Yes	Yes

a – clonal complex (CC); singleton (*); b – penicillin G (PG), erythromycin (Ery), clindamycin (Da), tetracycline (Tet), and trimethoprim sulfamethoxazole (SXT); c – ComD2 had an E151K substitution and ComD4 had an M77I and an E151K substitutions, both outside the sensor domain of ComD; nd – not determined.

### Candidate core genome

To determine if the genome content of NT strains is comparable to that of encapsulated strains, 34 NT representing the diversity of profiles identified by PFGE, MLST, and characterisation of the capsular region, were characterised by CGH using an array that covers the genome of nine encapsulated pneumococcal strains and R6 (a non-encapsulated derivative of D39) (Additional file 1). From the 3,052 genes present in the array, 1,666 (54.6%) were present in all NT tested, 839 (27.5%) were present in some, and 547 (17.9%) were absent in all (Additional file 2). In an independent analysis, conducted in the framework of an ongoing study, 180 encapsulated strains were analysed by CGH. These strains were representative of 20 serotypes and included all strains in the array (except R6). Results from this analysis were used for comparison. In this collection, 1,654 genes (54.2%) were present in all strains, the same proportion found for the NT isolates. Of these 1,654 genes, 1,499 (90.6%) were also present in all NT isolates (Additional file 2). Among the remaining 155 genes, 149 were present in some (but not all) NT and only 6 were absent in all. The proportion of these 155 genes present in the NT strains ranged between 80.0% and 58.7% (Additional file 3). The 149 genes with variable presence among NT strains could be grouped into the following functions: 22.8% cellular metabolism, 16.1% transporters, 8.7% DNA metabolism, 7.4% phages and mobile elements, 2.0% surface proteins, 2.0% signalling and communication, and 41.0% were annotated as hypothetical proteins. The six genes absent in all NT were SP_0346 (annotated as capsular polysaccharide biosynthesis protein Cps4A), SP_0368 (cell wall surface anchor family protein), SP_1153 (hypothetical protein), SP_2157 (alcohol dehydrogenase, iron-containing), SP_2158 (L-fucose isomerase), and SP_2168 (fucose operon repressor, putative).

Furthermore, NT isolates contained between 2,049 and 2,120 genes detected by CGH with an average of 2,095 genes, while the 180 encapsulated strains had between 2,119 and 2,306 genes with an average of 2,235. Based on these experiments, although the size of “core” genomes of NT versus encapsulated strains was comparable, NT strains characterised in this study had 6.3% less genes detected by CGH than encapsulated strains.

### Accessory regions (ARs)

**Figure 3:**
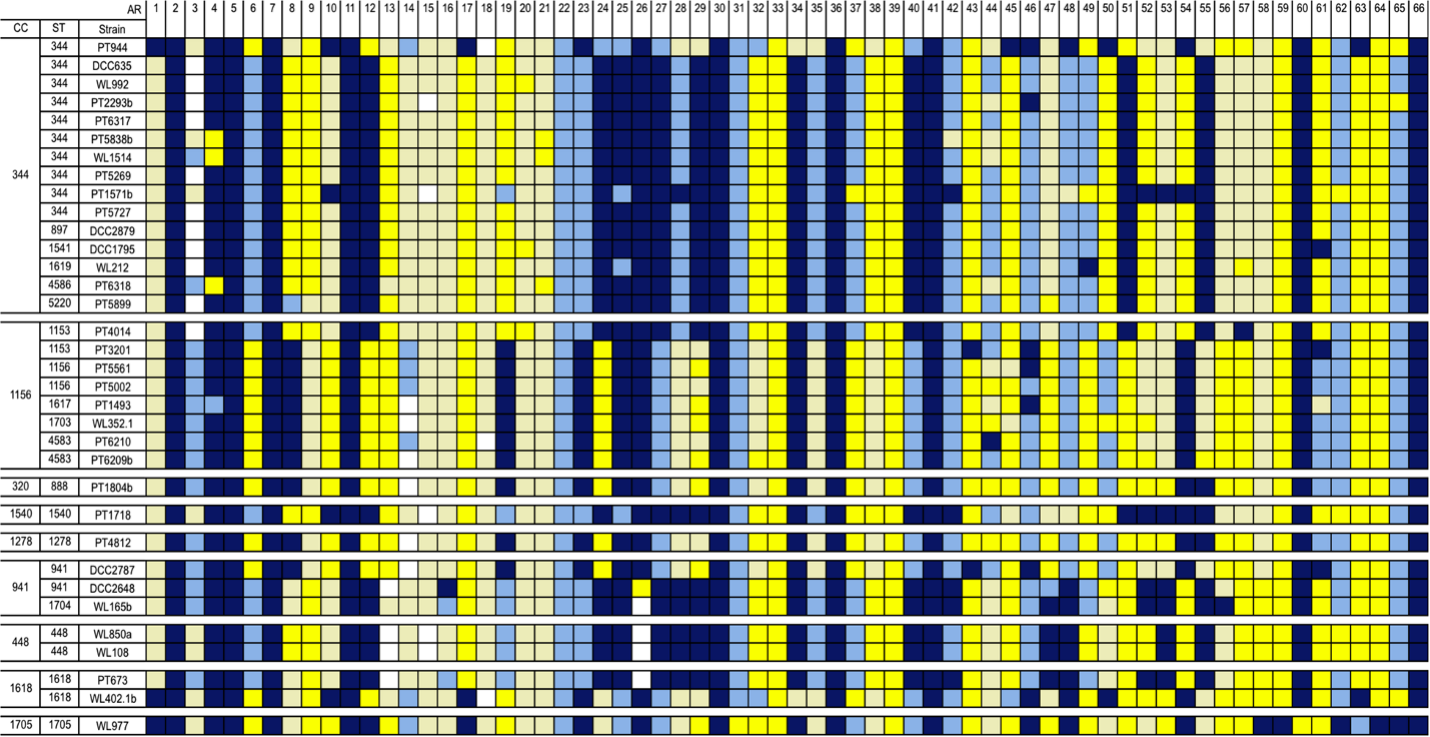
**Distribution of accessory regions (ARs) among NT strains.** Dark yellow – AR is present; light yellow – more than 50% of the genes in the AR are present; white – 50% of the genes in the AR are present; light blue – more than 50% of the genes in the AR are absent; dark blue – AR is absent.

To further analyse the genome content of NT strains, the presence of previously identified accessory regions was investigated (Figure 3) [19]. Of the 41 accessory regions described to date, 17 were present or partially present in all NT strains analysed (ARs 3, 6, 9, 13–15, 18, 20–22, 31–33, 35, 37–39) and 7 were absent in all (ARs 2, 5, 7, 11, 30, 36, and 41). Furthermore, 8 ARs were present, or at least partially present, in most strains (ARs 1, 8, 10, 16, 17, 19, 23, 28) and 9 ARs were absent, or mostly absent, in most strains (ARs 4, 12, 24–27, 29, 34, and 40).

**Figure 4:**
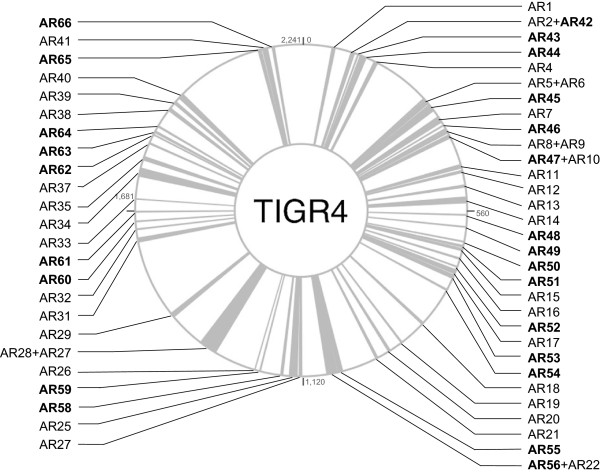
**Distribution of 66 accessory regions (ARs) over the TIGR4 genome.** Bold – new ARs identified in NT strains.

Twenty-five new ARs (named ARs 42 to 66), totalling 134 genes, were identified in this study. Their predicted functions are described in Table 2 and include ABC transporters, type II restriction-modification systems, phosphotransferase systems and proteins involved in metabolism, cell envelope, transport, and transcription regulation. These 25 ARs were dispersed around the TIGR4 genome (Figure 4). Of these, 22 ARs were present, or at least partially present, in most strains (ARs 42–54, 56–59, and 61–65), 2 ARs were absent, or mostly absent, in most strains (ARs 55 and 60), and AR66 (encoding for hypothetical proteins) was absent in all.

**Table 2:** **New accessory regions found in NT strains**

Accessory region	TIGR4 locus	Identified by STM ^a^	Predicted function ^b^
42	SP_0115-0117	Yes	Cell envelope
43	SP_0124-0126	No	Hypothetical
44	SP_0130-0144	Yes	ABC transporter (glucose)
45	SP_0314-0330	Yes	PTS system
46	SP_0367-0369	No	Cell envelope
47	SP_0391-0393	No	Cell envelope
48	SP_0569-0571	Yes	Type II RM system
49	SP_0595-0597	Yes	Hypothetical
50	SP_0627-0629	No	Hypothetical
51	SP_0636-0640	No	ABC transporter
52	SP_0683-0685	No	Hypothetical
53	SP_0703-0711	No	ABC transporter (aa)
54	SP_0737-0740	No	Transport & transcription regulation
55	SP_1030-1040	Yes	ABC transporter (iron)
56	SP_1042-1045	Yes	Metabolic
57	SP_1119-1125	Yes	Metabolic (glycogen)
58	SP_1160-1165	No	Metabolic (acetoin)
59	SP_1209-1211	No	Hypothetical
60	SP_1656-1658	No	Hypothetical
61	SP_1677-1679	No	Hypothetical
62	SP_1849-1851	No	Type II RM system
63	SP_1855-1859	Yes	Transport & transcription regulation
64	SP_1869-1872	Yes	ABC transporter (iron)
65	SP_2147-2154	No	Metabolic (arginine)
66	SP_2178-2183	Yes	Hypothetical

a – gene(s) within region(s) identified by signature-tagged mutagenesis as required for invasive disease [20]; b – ATP-binding cassette (ABC); phosphotransferase (PTS); restriction modification (RM); amino acid (aa).

Altogether, when looking for ARs absent in all NT, these were found to encode for capsular genes (AR7), type-I pili (AR11), sucrose ABC transporter (AR36), fucose metabolism (AR41), a putative bacteriocin (AR2), and several hypothetical proteins (ARs 5, 30 and 66).

### Virulence factors

A total of 496 of the genes present on the array were identified as virulence factors of pneumococcus based on published data (annotated in Additional file 2) [20–30]. Of these, 363 (73.2%) were present in all NT strains and 36 (7.3%) were absent in all. This latter group included genes associated with capsular synthesis (TIGR4 *cpsA*, *cpsC*, *cpsD*, *cpsE*, *cpsF*, and *cpsJ*), pilus islet-1, the virulence proteins *cbpA/pspC*, *pspA*, *nanE*, *glf*, and *ntpK* among others (Additional file 2). PCR analysis of pilus islet-1 and -2 confirmed the absence of these loci in all 52 strains.

Regarding competence-associated genes (n = 22), all were present in all strains, including the recently described *comG* operon (SP_1808 and SP_2047-53), encoding for a type-IV transformation pilus (Table 3) [31]. In addition, *comC* and *comD* alleles were determined by PCR for the 52 NT strains included in this study and a clear distinction between CCs could be observed for *comCD*: CCs 344, 448, and 941 encoded CSP2 and ComD2; CCs 320, 1156, 1278, 1618, and 1705 had CSP1 and ComD1; and CC1540 had CSP1 and ComD4 [32].

**Table 3:** **Virulence factors determined by CGH for NT clonal complexes**

Gene name and/or annotation	CC344 (n = 15)	CC1156 (n = 8)	CC320 (n = 1)	sing1540 (n = 1)	sing1278 (n = 1)	CC941 (n = 3)	CC448 (n = 2)	CC1618 (n = 2)	sing1705 (n = 1)
Competence proteins									
*comA*; competence factor transporting ATP-binding/permease protein ComA	1	1	1	1	1	1	1	1	1
*comB*; competence factor transport protein ComB	1	1	1	1	1	1	1	1	1
*comD*; putative sensor histidine kinase ComD	1	1	1	1	1	1	1	1	1
*comE*; response regulator ComE	1	1	1	1	1	1	1	1	1
*comX1*; transcriptional regulator ComX1	1	1	1	1	1	1	1	1	1
competence damage-inducible protein A	1	1	1	1	1	1	1	1	1
*coiA*; competence protein CoiA	1	1	1	1	1	1	1	1	1
competence protein ComF, putative	1	1	1	1	1	1	1	1	1
*celA*; competence protein CelA	1	1	1	1	1	1	1	1	1
*celB*; competence protein CelB	1	1	1	1	1	1	1	1	1
*ccs1*; competence-induced protein Ccs1	1	1	1	1	1	1	1	1	1
*ccs4*; competence-induced protein Ccs4	1	1	1	1	1	1	1	1	1
*ccs16*; competence-induced protein Ccs16	1	1	1	1	1	1	1	1	1
*cspC*-related protein, authentic point mutation	1	1	1	1	1	1	1	1	1
*pilD*; type IV prepilin peptidase, putative	1	1	1	1	1	1	1	1	1
*comGA/cglA*; competence protein CglA	1	1	1	1	1	1	1	1	1
*comGB/cglB*; competence protein CglB	1	1	1	1	1	1	1	1	1
*comGC/cglC*; competence protein CglC	1	1	1	1	1	1	1	1	1
*comGD/cglD*; competence protein CglD	1	1	1	1	1	1	1	1	1
*comGE*	1	1	1	1	1	1	1	1	1
*comGF*	1	1	1	1	1	1	1	1	1
*comGG*	1	1	1	1	1	1	1	1	1
Choline-binding proteins									
*cbpA*/*pspC*; choline binding protein A	0	0	0	0	0	0	0	0	0
*cbpD*; choline binding protein D	1	1	1	1	1	1	1	1	1
*cbpE*/*pce*; choline binding protein E	1	1	1	1	1	1	1	1	1
*cbpF*; choline binding protein F	0.1	0.9	1	0	1	0.3	0	0	1
*cbpG*; choline binding protein G	0.9	1	1	1	1	1	1	1	1
*lytA*; autolysin	1	1	1	1	1	1	1	1	1
*lytB*; endo-beta-N-acetylglucosaminidase	1	1	1	1	1	1	1	1	1
*lytC*; beta-N-acetylhexosaminidase	1	1	1	1	1	1	1	1	1
*pspA*; pneumococcal surface protein A	0	0	0	0	0	0	0	0	0
*pcpA*; choline binding protein PcpA	0.9	0.1	0	0	0	0	0	0	0
Colonisation-associated proteins									
*hyl*; hyaluronidase	0.9	1	1	1	1	1	1	0.5	1
*nanA*; neuraminidase A/siliase A precursor	0.1	0	0	1	0	0	0	0.5	1
*pavA*; adherence and virulence protein A	1	1	1	1	1	1	1	1	1
*rlrA*; transcriptional regulator, putative	0	0	0	0	0	0	0	0	0
*bgaA*; beta-galactosidase	0.1	0.9	1	0	1	0.3	0	0	0
*eno*; phosphopyruvate hydratase	1	1	1	1	1	1	1	1	1
*pyrR*; bifunctional pyrimidine regulatory protein PyrR uracil phosphoribosyltransferase	1	1	1	1	1	1	1	1	1
*strH*; beta-N-acetylhexosaminidase	1	1	1	1	1	1	1	1	1
*trpG*; anthranilate synthase component II	1	1	1	1	1	1	1	1	1
*phoU*; phosphate transport system regulatory protein PhoU, putative	0.1	0.9	1	1	1	0.3	0	0.5	0
*rr01*; DNA-binding response regulator	1	1	1	1	1	1	1	1	1
transcriptional regulator SPY2053	1	1	1	1	1	1	1	1	1
Other major virulence factors									
*ply*; pneumolysin	1	1	1	1	1	1	1	1	1
*psaA*; manganese ABC transporter, manganese-binding adhesion lipoprotein	1	1	1	1	1	1	1	1	1
*htrA*; serine protease	1	1	1	1	1	1	1	1	1
*IgA*; immunoglobulin A1 protease	1	1	1	1	1	1	1	1	1
*spxB*; pyruvate oxidase	1	1	1	1	1	1	1	1	1
*piaA*; iron-compound ABC transporter, iron compound-binding protein	0.1	0.9	0	0	0	0.3	0	0.5	1
*piaB*; iron-compound ABC transporter, permease protein	0.1	0.9	0	0	0	0.3	0	0.5	1
*piaC*; iron-compound ABC transporter, permease protein	0.1	0.9	0	0	0	0.3	0	0.5	1
*piaD*; iron-compound ABC transporter, ATP-binding protein	0.1	0.9	0	0	0	0.3	0	0.5	1
*piuA*; iron-compound ABC transporter, iron-compound-binding protein	1	1	1	1	1	1	1	1	0
*piuB*; iron-compound ABC transporter, permease protein	1	1	1	1	1	1	1	1	0
*piuC*; iron-compound ABC transporter, permease protein	1	1	1	1	1	1	1	1	0
*piuD*; iron-compound ABC transporter, ATP-binding protein	1	1	1	1	1	1	1	1	0
*zmpB*; zinc metalloprotease	0	0.8	0	0	0	0.3	0	0	1

CC – clonal complex; sing – singleton; numbers between 0 and 1 indicate the relative proportion of strains containing the gene.

Nine choline binding proteins have been implicated in virulence, and all were present on the array [20, 27, 33, 34]. Of these, *cbpD*, *cbpE/pce*, *lytA*, *lytB*, and *lytC* were present in all strains, with *cbpA/pspC* and *pspA* being absent in all strains. Variation between CCs was found for *cbpF, cbpG* and *pcpA* (Table 3).

In addition, 12 genes implicated in colonisation were present on the array. Of these, *pavA, eno, pyrR*, *strH*, *trpG, rr01*, and *SPY2053* were present in all NT, while *rlrA* was absent in all strains. Clonal variation was found for genes *hyl*, *nanA*, *bgaA,* and *phoU* (Table 3).

Among other major virulence factors, *ply*, *psaA, htrA, IgA*, and *spxB* were present in all strains with variations between clones found for the operons *piuA-D* and *piaA-D* and *zmpB*.

Further details on the variable presence of virulence genes can be found in Additional file 2.

### Intraclonal variation

**Figure 5:**
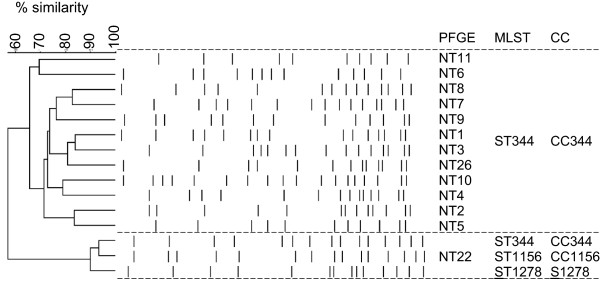
**Comparison of PFGE patterns found for clonal complex (CC) 344, CC941, CC448, and CC1156.** Dendrogram generated by UPGMA and Dice similarity with an optimisation of 1% and a tolerance of 1.5%. CC – clonal complex; S – singleton.

**Figure 6:**
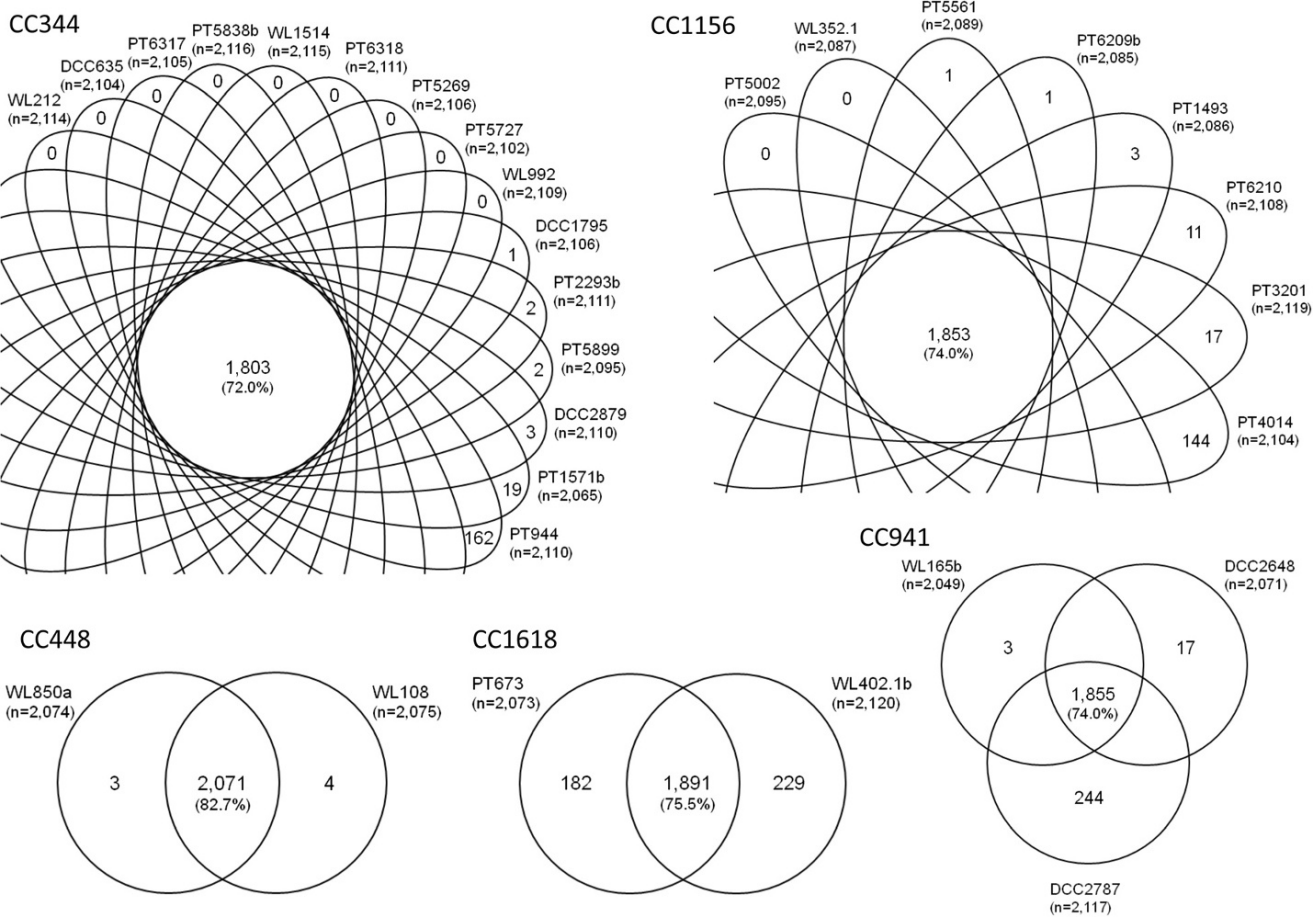
**Intraclonal diversity of NT strains.** CC – clonal complex; numbers in the centre represent the number of genes shared by all strains of a given CC/singleton and the percentage in relation to the total number of genes detected for NT; other numbers represent the number of genes found exclusively for a given strain in comparison with strains from the same CC.

Comparison of SmaI-PFGE patterns of NT strains resulted in an unexpected high diversity of profiles for strains belonging to the same ST (Figure 5) [5]. Likewise, there were also strains with similar PFGE profiles belonging to different STs. This lack of concordance was puzzling, as previous studies have found a good general agreement with PFGE and MLST for encapsulated pneumococci [35]. To investigate possible genomic variations that could account for the lack of concordance found between PFGE and MLST results, CGH results were compared for strains belonging to the same CC. For any given CC, all strains analysed shared at least 72% of the genes detected in the NT pool (Figure 6).

When we looked at intraclonal diversity, within each CC, variation between strains was mostly due to only a few (if any) genes. Still, exceptions were found: strains PT944 of CC344, PT4014 of CC1156, and DCC2787 of CC941 had 162, 144, and 244 genes, respectively, uniquely present in their genomes compared to other strains of the same CC. Also, the two strains of CC1618 were found to differ from each other in more than 400 genes.

When looking for the functions of genes uniquely present in one strain of a given CC, most were found to encode for hypothetical proteins (51.3%). Other genes had the following functions: transport and secretion (13.4%), cell metabolism (9.9%), phages and mobile elements (9.5%), DNA metabolism (7.8%), cell wall, cell membrane, and cell division (3.8%), signalling and communication (2.7%), and stress (1.5%). Furthermore, only 10.2% of this latter group of genes have been described as virulence genes. Not surprisingly, close to half of these genes were found in ARs (44.4%).

**Figure 7:**
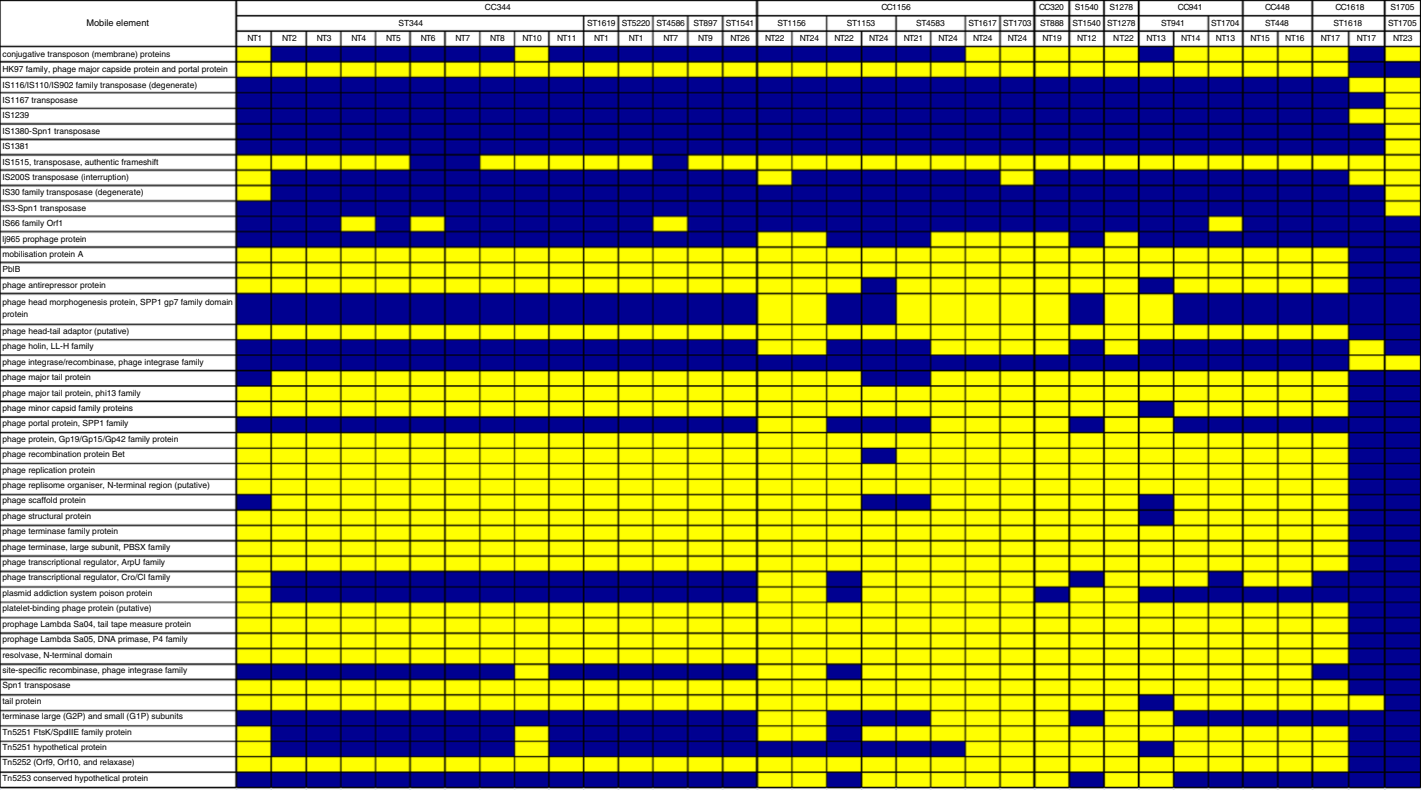
**Intraclonal variability of mobile elements.** NT1 to NT24 refer to PFGE patterns. Yellow – present; blue – absent.

To investigate if the high variability of PFGE types found could be due to the presence of prophages, as previously reported [36], or the presence of other mobile elements, we evaluated their distribution among NT strains (Figure 7). In some cases, e.g. NT1, NT2, and NT6 of ST344 or NT22 and NT24 of ST1153, the content of mobile elements was indeed distinct between strains, which might explain the variability found. However, in other cases, such as NT2, NT3, NT5, NT8, and NT11 of ST344 and NT15 and NT16 of ST448, the strains shared the same mobile elements. On the other hand, examples of strains belonging to the same PFGE type and ST but with different mobile elements’ profiles were also observed (e.g. NT17 of ST448). To complement this analysis, the presence of prophages was also determined by *lytA* hybridisation (Additional file 4). In ST344, the six PFGE types tested exhibited three *lytA* hybridisation patterns, whereas the two ST448 PFGE types tested showed the same *lytA* hybridisation pattern. According to these results, the high variability of PFGE types observed within STs could not be entirely explained by the presence of prophages or other mobile elements.

## Discussion

In this study we aimed to characterise the genomic content of a collection of NT strains representative of the carriage lineages circulating in Portugal in a period of 11 years (1997–2007). Strains were analysed by CGH against a panel of 10 pneumococcal strains and their capsular region was sequenced. According to their capsular regions, strains in this study could be classified as NCC2, as they all contained *aliB*-like genes [15]. Strains with similar capsular regions have also been identified in carriage and disease isolates circulating in Switzerland, the Netherlands, UK, USA, Brazil, South Korea, Thailand, and the Gambia [15–17, 37]. In our collection we did not find isolates of *cps* type NCC1 (containing the *pspK/nspA* gene) and we did not include NT strains derived from encapsulated lineages that had alterations in the capsular operon leading to absence of capsular production (Group I NT).

Of interest, a recent study by Park, *et al*. aimed to characterise invasive NT strains from the USA. The authors reported that these strains are rare, accounting for less than 1% of the invasive pneumococcal disease cases, and most are of Group I NT, with only a few cases caused by NCC2 NT. Nonetheless, it has been clearly demonstrated that NCC2 NT are capable of causing invasive disease and therefore should not be disregarded [17, 37].

In relation to core genome, 54.6% of the genes represented on the array were found in all NT strains, the same proportion found for a collection of 180 encapsulated strains used for comparison (54.2%). However, the average number of total genes detected in the NT strains (2,095) was 6% less than the corresponding value found for encapsulated strains. Still, this result should be interpreted with caution as, by using a CGH approach, NT genes were probably missed to an unknown extent.

Twenty-five new ARs, dispersed around the TIGR4 genome, were identified in this study. Of the 66 ARs identified to date, only seven were absent in all NT and encoded for genes associated with sugar metabolism, capsular synthesis, type-I pilus, and hypothetical proteins [19]. Also, more than 90% of the virulence factors identified in pneumococcus were found in NT. The most relevant virulence factors absent from all NT were the capsular genes and type-I pilus (referred to above), type-II pilus, choline-binding protein A (*cbpA*/*pspC*), and pneumococcal surface protein A (*pspA*) [23]. Also absent in the majority of NT was the major iron ABC transport system *piaA-D*. However, *piuA-D*, a second iron ABC transport system, was present in the majority of NT. Mutations in these systems have been shown to result in mild (*piuA-D*) to moderate (*piaA-D*) reduction in virulence [38]. Together with the lack of capsule and other important virulence genes, the absence of these genes in NT should contribute to a lower propensity of NT to cause disease.

As expected, all strains had all competence genes, including the newly described transformation pilus [14, 31, 39]. According to the type of competence stimulating peptide (CSP, encoded by *comC*) secreted by pneumococcal strains, strains can be divided in pherotypes. The dominant pneumococcal pherotypes are CSP1 and CSP2, respectively found in 60-75% and 25-40% of carriage or clinical isolates [40, 41]. In NT, the dominant pherotype was CSP2 (65% of the strains), with the remaining strains belonging to pherotype CSP1. In our study, pherotype was a clonal property, with all strains within a CC belonging to the same pherotype. The same association was previously observed in encapsulated pneumococcus [42]. These results further support that NT are *bona-fide* pneumococci, in contrast with atypical strains of ambiguous speciation, where multiple ComC alleles can be found [43].

To explore the reasons underlying the observation that NT had highly variable PFGE profiles in contrast to relatively conserved STs, we assessed whether the presence of prophages or other mobile elements could account for these observations. Although that seemed to be the case in some strains, the presence of these mobile elements could not entirely explain the variability found in NT isolates, at least with the approaches that were used. A more detailed characterisation of phage presence, such as the prophage typing system proposed by Romero, *et al.*, could have provided additional information but was beyond the purpose of this study [44, 45].

Our study has a major limitation. Information obtained by CGH is restricted to what is present in the array and therefore limited by nature. Still, interesting information regarding variability and presence/absence of pneumococcal genes implicated in virulence was obtained, providing further hypothesis related to the low disease capacity of these strains. Our study has also some strengths. The thorough characterisation of a representative collection of NT circulating in Portugal for over a decade provided insight on the most frequent features of the lineages in circulation and definitely supported the inclusion of these strains as part of the pneumococcal population.

## Conclusions

NT circulating in Portugal are a homogeneous group belonging to *cps* type NCC2. Our observations support that this group are *bona-fide* pneumococcal isolates that do not express the capsule but are otherwise essentially similar to encapsulated pneumococci, having a comparable core genome and most virulence factors. Given that NT are not targeted by current pneumococcal vaccines and that they are highly transformable, we recommend that these isolates are routinely identified and reported in surveillance studies monitoring pneumococcal serotype evolution.

## Methods

### Ethics statement

Approval for the original studies [5, 46, 47] was obtained from the Ministry of Education. The studies were registered and approved at the Health Care Centre of Oeiras that reports to Administração Regional de Saúde (ARS; “Regional Health Administration”) of Lisboa and Vale do Tejo from the Ministry of Health. Signed informed consent was obtained from parents/guardians of participating children. All samples were coded numerically upon collection and processed anonymously. In the present study, only bacterial isolates were characterised (no human subjects, human material or human data were used). Thus, ethical approval was not required.

### Study collection

We selected 52 NT strains for detailed characterisation. This collection was extracted from a total of 422 NT strains isolated between 1997 and 2007 from the nasopharynx of preschool children attending day-care centres in Lisbon, Portugal. The isolates were previously characterised by PFGE, MLST, and antibiotic susceptibility to penicillin, amoxicillin, ceftriaxone, erythromycin, clindamycin, tetracycline, chloramphenicol, and trimethoprim sulfamethoxazole (SXT) [5, 46, 47]. The 52 strains characterised in this study were selected to cover the diversity of profiles observed among the 422 isolates, as determined by PFGE, MLST and antibiotyping. CCs were defined based on goeBURST classification [48].

### DNA extraction

Total genomic DNA was isolated using either the DNeasy Blood & Tissue kit (Qiagen, Hilden, Germany), or the High Pure PCR Template Preparation kit (Roche Diagnostics GmbH, Mannheim, Germany), according to the manufacturers' recommendations.

### Characterisation of the capsular (*dexB-aliA*) region

The *dexB-aliA* region, corresponding to the capsular region in encapsulated pneumococci, was amplified by PCR using the primers described by Kilian, *et al.* using the following conditions: 92°C for 2 min; 30 cycles of 92°C for 10 sec, 58°C for 30 sec, and 68°C for 15 min; and a final extension at 68°C for 7 min [49]. For a final volume of 50 μL, the PCR mixture contained 20 ng of DNA, 1x Expand Long Template buffer 3 with 2.75 mM MgCl_2_ (Roche), 3.2 mM (each) deoxynucleoside triphosphates, 0.4 mM of each primer, and 3.75U of Expand Long Template enzyme mix (Roche). Amplicons were purified using ExoSAP by incubating 30 μL of the PCR product with 6U of Exonuclease I (New England Biolabs, Ipswich, MA, USA) and 6U of Shrimp Alkaline Phosphatase (GE Healthcare, Waukesha, WI, USA) for 30 min at 37°C followed by 15 min at 80°C.

RFLP signatures of the capsular region were determined after digestion of 15 μL of purified PCR fragments with HinfI or StyI for 3 h at 37°C. For a total volume of 20 μL, 5U of enzyme, 1x NEBuffer (New England Biolabs), and 2 μg of BSA (for StyI) were added. Results were analysed by gel electrophoresis and Bionumerics software (version 3.0, Applied Maths, Gent, Belgium). Patterns were clustered by UPGMA and a dendrogram was generated from a similarity matrix calculated using the Dice similarity coefficient with an optimisation of 0.5% and a tolerance of 1.0%. RFLP patterns determined by digestion with HinfI were arbitrarily named A to H.

Sequencing of the capsular region of representative RFLP patterns was performed by primer walking. Primers were designed using the nucleotide sequence of strain 110.58 as a template [GenBank:AY653211.1] (Additional file 5) [17, 49]. PCR products were obtained, purified, and sent to Macrogen, Inc. (Seoul, South Korea) for sequencing. Additional primers were designed to amplify and sequence the gaps between fragments as needed. Sequences were analysed and aligned using the Lasergene software (DNASTAR Inc., Madison, WI, USA). Nucleotide sequences of the capsular region were further analysed by performing a nucleotide BLAST search at the National Center for Biotechnology Information Website against the nucleotide database and also against the capsular region sequences previously described for NT strains [15–17, 50].

### CGH

Microarrays used in this study were 12x135K NimbleGen arrays (Roche). Labelling, hybridisation, and washing of the samples was done as recommended by the manufacturer using a NimbleGen microarray workflow (Roche): 1 μg of DNA from each strain was fluorescently labelled with Cy3 Random Nonamers using the NimbleGen One-Color DNA Labeling kit, samples were hybridised to the microarray slide using the NimbleGen Hybridization System, slides were washed using the NimbleGen Wash Buffer kit, and CGH data was acquired on a NimbleGen MS 200 Scanner. Normalisation and background correction of data was done by quantile RMA analysis using the ArrayStar software (DNASTAR). A cut-off of 512 was reached by drawing a graph of frequencies of signal intensities for all strains. Genes with signal intensities of 512 or above were considered present (assigned 1) and genes with signal intensities bellow that value were considered absent (assigned -1) from a given strain.

### Validation of the microarray

The microarray used was designed based on the genome sequence of 10 pneumococcal strains: TIGR4, R6, D39, BHN100, CBR206, LGST215, BHN191, BHN418, Sp14-BS69, and Sp3-BS71 [51–58]. Triplicates of probes representing genes present in these strains were added sequentially resulting in 3,052 non-redundant ORFs. Nine of the 10 strains represented in the array were hybridised with it for validation. Only 16 of 3,052 (0.52%) ORFs present in the microarray gave false negative results (Additional file 6). Most of these genes encoded for hypothetical proteins or mobile elements that might have been lost (during repeated handling). None of the 16 genes were part of the core genome, were related to virulence or located in ARs.

### ARs

The presence of ARs (or regions of diversity) previously identified (reviewed in [19]) was investigated for NT strains. New ARs were identified as defined by Tettellin and Hollingshead: three or more contiguous genes in the TIGR4 genome that were absent from at least one of the analysed strains [59]. Classification of new ARs followed the nomenclature proposed by Blomberg, *et al*. and was done sequentially [59].

### Detection and characterisation of genes by PCR

The presence of genes *comC*, *comD*, and *piaA* and the presence of type-I and type-II pili was assessed by PCR and characterised by sequencing when needed. *ComD* was amplified using primers comD_F (ATTAAAGGTGGGGAGATGAGG) and comD_R (CCAGCATAATCATGTCG), designed with TIGR4 [GenBank:NC_003028.3) and R6 [GenBank:NC_003098.1] nucleotide sequences as templates. Amplicons with an expected size of 841 bp were amplified using the following conditions: 94°C for 4 min; 30 cycles of 94°C for 30 sec, 55°C for 30 sec, and 72°C for 1 min; and a final extension at 72°C for 4 min. For a final volume of 50 μL, the PCR mixture contained 1 μL DNA, 1x Colorless GoTaq Flexi buffer (Promega, Madison, WI, USA), 2.5 mM MgCl_2_, 80 μM (each) deoxynucleoside triphosphates, 0.4 mM of each primer, and 2.5U of GoTaq DNA polymerase. Amplicons were purified using ExoSAP as described above, sent to Macrogen for sequencing, and analysed by using Lasergene software. The presence of *comC* was assessed as described by Whatmore, *et al*. or Carrolo, *et al.* [40, 60]; the presence of *piaA* was assessed as described by Whalan, *et al.* [61], and the presence of type-I and type-II pili as described by Zahner, *et al.* [62].

### Prophage detection by southern hybridisation of PFGE restriction profiles with a *lytA*probe

Preparation of chromosomal DNA, digestion with SmaI endonuclease, and separation of DNA fragments by PFGE were carried out as previously described [63]. Southern blotting of PFGE gels with a probe for the *lytA* gene was performed as previously described [36].

## Availability of supporting data

Microarray data supporting the results of this article have been submitted to NCBI Gene Expression Omnibus (GEO) archive repository [64]. The GEO Series Accession Number is GSE58329.

## Electronic supplementary material

Additional file 1: **Strains represented in the array.** (PDF 85 KB)

Additional file 2: **Core genome, virulence genes, and accessory regions.** a – annotations for: TIGR4 (SP_), D39 (SPN_), R6 (spr), CBR206 (CBR206_), LGST215 (DCCPN215_), Sp3-BS71 (SP3_), Sp14-BS69 (SP14_), BHN100 (BHN100_), BHN191 (BHN191_), and BHN418 (BHN418_); Red – genes present in all NT strains analysed; bold – new accessory regions identified in NT strains. (XLSX 530 KB)

Additional file 3: **Percentage of the 155 genes absent in some NT but present in a group of 180 diverse encapsulated strains (see text).** ST – multi-locus sequence type; CC – clonal complex. (PDF 41 KB)

Additional file 4: **Detection of prophages by** ***lytA*** **hybridisation.** A – SmaI-PFGE patterns of strains representing ST344 and ST448; B – southern blotting of the PFGE gel with a probe for *lytA*. (PDF 6 MB)

Additional file 5: **Primers used to amplify the capsular region of NT strains.** (PDF 93 KB)

Additional file 6: **Validation of the microarray.** a – R6 is a derivative of D39 and was not hybridised. (PDF 13 KB)

## Abbreviations

AR: Accessory region
CC: Clonal complex
CGH: Comparative genomic hybridisation
MLST: Multi-locus sequence typing
NT: Non-typeable pneumococcus
PFGE: Pulsed-field gel electrophoresis
RFLP: Restriction fragment length polymorphism
ST: Multi-locus sequence type
SXT: Trimethoprim sulfamethoxazole.
