# Supplementary material for: Non-typeable pneumococci circulating in Portugal are of cps type NCC2 and have genomic features typical of encapsulated isolates
Source: BMC Genomics. 2014 Oct 6;15(1):863. doi: 10.1186/1471-2164-15-863 (PMC4200197; doi:10.1186/1471-2164-15-863)
Supplement: Supplementary file 1 — Additional file 1: Strains represented in the array. (PDF 85 KB) [file 12864_2014_6549_MOESM1_ESM.pdf]

# Additional file 1

| Strain | Serotype | MLST | Reference |
|---|---|---|---|
| **TIGR4** | 4 | 205 | [50] |
| **D39** | 2 | 595 | [49] |
| **R6** | - | 595 | [48] |
| **CBR206** | 19F | 179 | [54] |
| **LGST215** | 19F | 179 | [55] |
| **Sp3-BS71** | 3 | 180 | [52] |
| **Sp14-BS69** | 14 | 124 | [52] |
| **BHN100** | 19F | 162 | [53] |
| **BHN191** | 6B | 138 | [51] |
| **BHN418** | 6B | 138 | [51] |
