# Supplementary figures and images for: Non-typeable pneumococci circulating in Portugal are of cps type NCC2 and have genomic features typical of encapsulated isolates
Source: BMC Genomics. 2014 Oct 6;15(1):863. doi: 10.1186/1471-2164-15-863 (PMC4200197; doi:10.1186/1471-2164-15-863)

Additional file 3

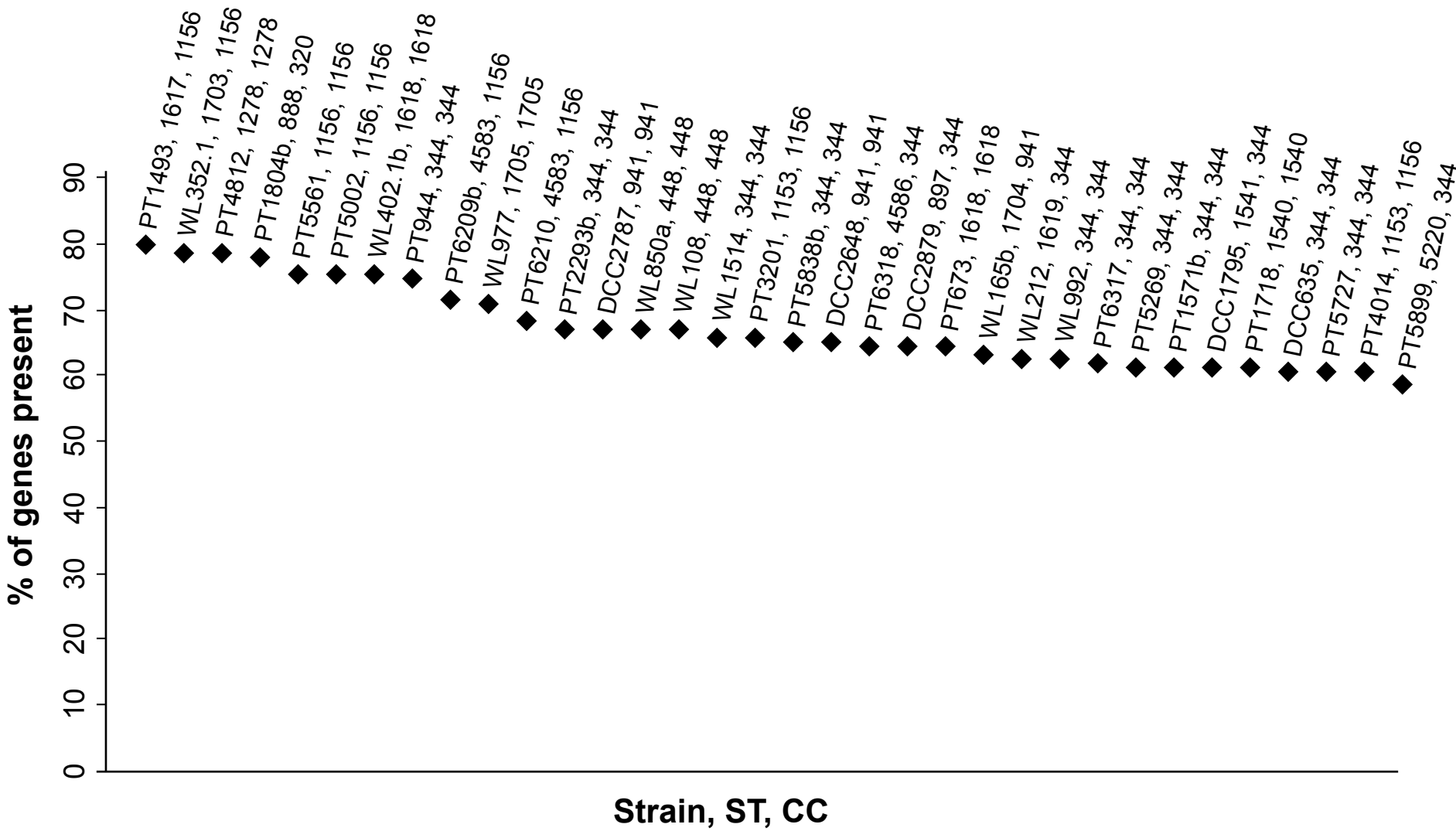

Supplement: Supplementary file 3 — Additional file 3: Percentage of the 155 genes absent in some NT but present in a group of 180 diverse encapsulated strains (see text). ST – multi-locus sequence type; CC – clonal complex. (PDF 41 KB) [file 12864_2014_6549_MOESM3_ESM.pdf]

Additional file 4

(A)

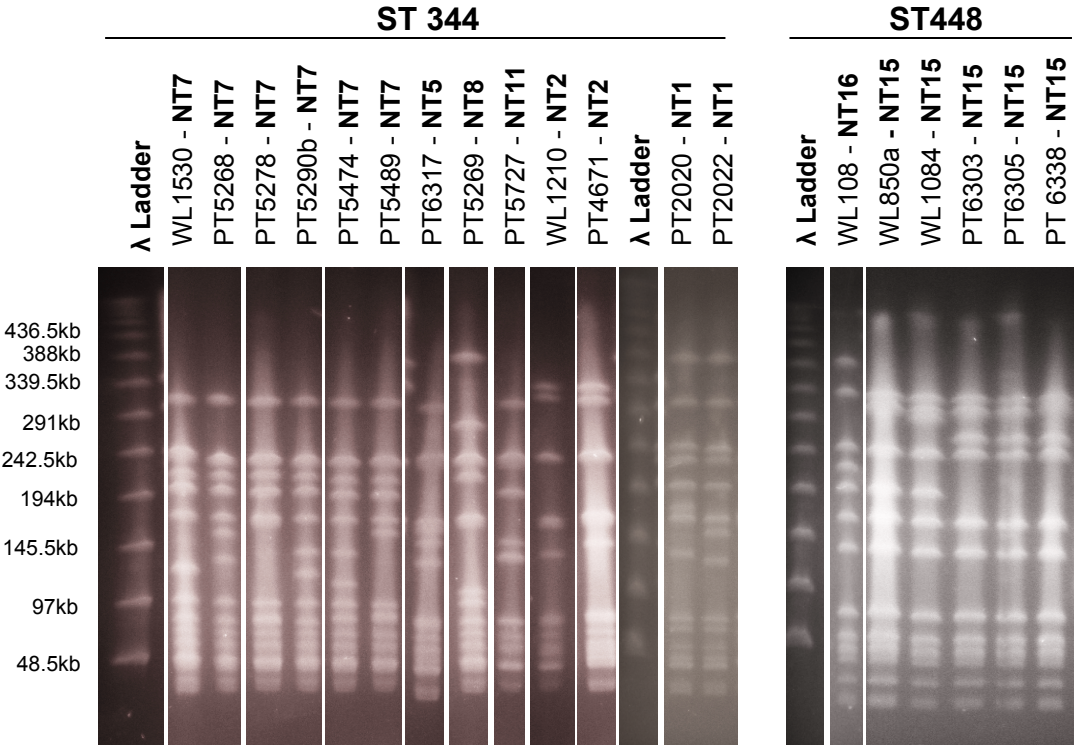

(B)

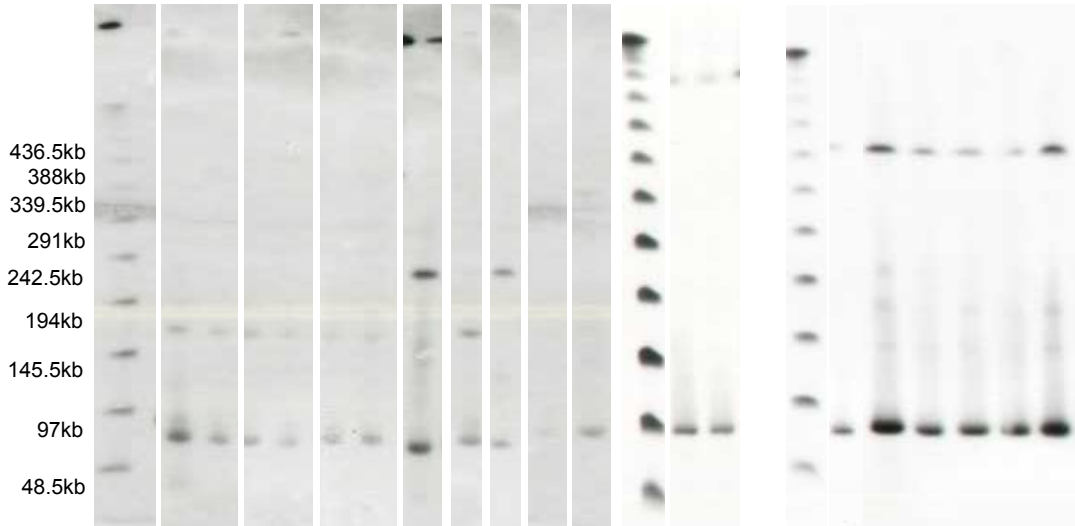

Supplement: Supplementary file 4 — Additional file 4: Detection of prophages by lytA hybridisation. A – SmaI-PFGE patterns of strains representing ST344 and ST448; B – southern blotting of the PFGE gel with a probe for lytA. (PDF 6 MB) [file 12864_2014_6549_MOESM4_ESM.pdf]
