# Supplementary material for: Non-typeable pneumococci circulating in Portugal are of cps type NCC2 and have genomic features typical of encapsulated isolates
Source: BMC Genomics. 2014 Oct 6;15(1):863. doi: 10.1186/1471-2164-15-863 (PMC4200197; doi:10.1186/1471-2164-15-863)
Supplement: Supplementary file 5 — Additional file 5: Primers used to amplify the capsular region of NT strains. (PDF 93 KB) [file 12864_2014_6549_MOESM5_ESM.pdf]

Additional file 5

Primer	Sequence	Reference
**cap F**	GTTKTGGCTAACTTGTCCAATG	[46]
**cps 1R**	TGAGCTGTATAGGCGTGGCG	this paper
**cps 1F**	AATCGCCACGCCTATACAGC	this paper
**cps 2R**	GATACTGAGCATTCGATTCC	this paper
**cps 2F**	AGCGGCCTTCAAAAAGTGGC	this paper
**cps 3R**	TACGGATGGTCAATTCTTGG	this paper
**cps 3F**	GACCAAGAATTGACCATCCG	this paper
**cps 4R**	ATCCAAGCCTGTGCTTCAGC	this paper
**cps 4F**	ATGCTGAAGCACAGGCTTGG	this paper
**cps 5R**	ATATGAGCCGTTTGCTCAGC	this paper
**cps 5F**	AAGATTGCTGAGCAAACGGC	this paper
**cps 6R**	GCAGCTAAAACACCAGCTGC	this paper
**cps 6F**	GCAGCTGGTGTTTTAGCTGC	this paper
**cap R**	CTGTCAACCAAGCTTGGGC	[46]
**cps 7F**	AAGTGGCTCTTAGGAGCAGG	this paper
**cps 7F_2**	GGTAAAATGTCAAGCGACCC	this paper
**cps 7R**	GAGTTGGCGCTCCGTAATCC	this paper
**cps 8F**	GTCTATAGTCCACAAGAGGC	this paper
**cps 9F**	TACCAAAGCTTATTCACAGG	this paper
**cps10F**	CACGCCCAGAACCTTACTGG	this paper
**cps 11R**	GCTTTCTTGCTCCCATTTGG	this paper
**cps 12F**	AGCTAATTACAAGGGTAGCC	this paper
**cps 12R**	AAAGGGTGGAAGGTCAGTCG	this paper
**cps 13F**	GTTAGAATACCGTAGTCTTCG	this paper
**cps 13R**	ACTGGTGTTGACGAGGTGGC	this paper
**cps 14R**	TTAGTTTCAATCACCGAAGC	this paper
**cps 15F**	GCTATCAACCATACGAGC	this paper
**cps 16F**	GGAAACAGCTAGTCTGTTGG	this paper
**cps 16R**	GCTTGCTCAAACCATTCAAGC	this paper
**cps 17F**	CAGAAGAGMAYYTMTGTTGGC	this paper
**cps 17F_2**	CAGAAGAGAACCTATGTTGGC	this paper
**cps 18F**	GTTAGCAAGTTCGTCTAAGG	this paper
**cps 18R**	TTCTGCATCTAGTAGGATGC	this paper
**cps 19F**	CCTCTAGCTAATTACAAGGG	this paper
**cps 19R**	TGATGATAAAGGGTGGAAGG	this paper
**cps 20F**	GTATTCTCTATAGCGGACC	this paper
**cps 20R**	GTGTGATTGTAAGCCTTACG	this paper
