# Supplementary material for: Non-typeable pneumococci circulating in Portugal are of cps type NCC2 and have genomic features typical of encapsulated isolates
Source: BMC Genomics. 2014 Oct 6;15(1):863. doi: 10.1186/1471-2164-15-863 (PMC4200197; doi:10.1186/1471-2164-15-863)
Supplement: Supplementary file 6 — Additional file 6: Validation of the microarray. a – R6 is a derivative of D39 and was not hybridised. (PDF 13 KB) [file 12864_2014_6549_MOESM6_ESM.pdf]

Additional file 6

Strain	No. ORFs in array	False negatives	Signal intensity	Description
**TIGR4**	2,238	SP_1217	459	Hypothetical protein
		SP_1921	442	Hypothetical protein
**R6**	113	Not hybridised[a]		
**D39**	13	None		
**BHN100**	192	BHN100_0544	392	Glutamyl aminopeptidase
		BHN100_1337	118	Caax amino protease
		BHN100_2111	12	Transposase
		BHN100_2148	361	Transposase
		BHN100_2155	348	Transposase
**CBR206**	170	CBR206_1142	282	Tn5252
		CBR206_1354	246	Hypothetical protein
		CBR206_2210	56	Hypothetical protein
		CBR206_2248	330	Phage holing
**LGST215**	4	None		
**BHN191**	178	BHN191_0760	446	EcoBI specificity protein
		BHN191_0783	91	Hypothetical protein
		BHN191_2196	6	Cell wall surface anchor family protein
**BHN418**	1	None		
**Sp14-BS69**	92	SP14_0054	361	Gp18
		SP14_1091	121	Hypothetical protein
**Sp3-BS71**	51	None		
Total ORFs	3,052			
